# Effect of Dentin-Disinfection Chemicals on Shear Bond Strength and Microhardness of Resin-Infiltrated Human Dentin in Different Adhesive Protocols

**DOI:** 10.3390/medicina58091244

**Published:** 2022-09-08

**Authors:** Satheesh B. Haralur, Mohammed Mudawi Alqahtani, Roaa Ali Alqahtani, Rayan Mohammed Shabab, Khalid Ali Hummadi

**Affiliations:** 1 Department of Prosthodontics, College of Dentistry, King Khalid University, Abha 62529, Saudi Arabia; 2 Interns, College of Dentistry, King Khalid University, Abha 62529, Saudi Arabia; mohmmad.282@hotmail.com (M.M.A.); Droaa.alq@outlook.sa (R.A.A.); Rayanshabab@hotmail.com (R.M.S.); znobagt1@gmail.com (K.A.H.)

**Keywords:** tooth disinfection, antimicrobial substances, shear bond strength, microhardness, chlorhexidine, sodium hypochlorite, EDTA, povidone iodine

## Abstract

*Background and Objectives*: Bacteria and its remnants beneath the restorations predispose the tooth to secondary caries and pulpal pathology. Hence, various chemical antibacterial agents are suggested to disinfect the prepared tooth structure before the definitive restorative procedure. This study aimed to investigate the effects of chemical disinfectant solutions on the micro-shear bond strength (µSBS) and microhardness of total-etch and self-etch resin-infiltrated human dentin. *Materials and Methods:* 100 caries-free intact permanent third molar teeth were vertically sectioned into the buccal and lingual half. All these specimens were mounted on acrylic resin and underlying dentin surfaces were exposed by grinding. Samples were randomly divided into five groups [*n* = 20] following total-etch and self-etch adhesive protocol. Teeth samples were divided according to surface treatment, as Group I (Control-CNT), Group II (2% chlorhexidine-CHX), Group III (5.25% sodium hypochlorite-NaOCl), Group IV (17% ethylenediaminetetraacetate acid—EDTA) and Group V (10% povidone iodine-PVI). A randomly selected 10 samples from each subgroup were used for µSBS and microhardness tests. After surface treatment and bonding procedure, nono-hybrid composite cylinders with a 3-mm diameter and 2-mm height were directly cured over the dentin substrate. The samples for µSBS were subjected to 5000 thermocycles and tested using a universal testing machine. Microhardness was assessed using a micro-indenter instrument, data were statistically analyzed using a one-way analysis of variance and Tukey HSD tests at *p* < 0.05. *Results:* Amongst the chemical disinfectant assessed, 2% CHX did not affect µSBS and produced a marginal reduction in dentin microhardness compared to the control group. The 5.25% NaOCl and 17% EDTA significantly compromised the microhardness of the dentin substrate. Meanwhile, 10% PVI surface treatment resulted in a substantial reduction in µSBS between composite and dentin. *Conclusions:* CHX with preservation of bonding to dentin and insignificant negative effect on dentin microhardness is a safe option for tooth disinfection.

## 1. Introduction

The bacterial remnants after tooth preparation could survive and multiply. It can potentially lead to recurrent caries, pulpal damage, and eventual failure of the restoration [1]. Researchers demonstrated the survival of bacteria beneath the restoration for more than a year [2]. Complete removal of carious dentin by mechanical methods could lead to compromised pulp health and structural integrity of the tooth. Complete elimination of bacteria from the prepared teeth surface is not feasible despite the use of disclosing agents. Hence, in contemporary dentistry, chemical disinfection of prepared teeth has become an important step before placing restorative material. Besides the elimination of viable bacteria and their toxins from the restorative interface, it also reported it impedes the long-term deterioration of bond strength with inhibition of matrix metalloproteinases [MMPs] activity [3]. Disinfection of prepared dentin surface is additionally essential in the self-etch adhesive protocol, wherein lack of irrigation step of etched dentin results in incomplete removal of the bacteria-embedded smear layer. Currently, various antimicrobial agents are utilized for the disinfection of prepared teeth, including chlorhexidine (CHX), sodium hypochlorite, iodine, EDTA, fluoride-based solutions, and benzalkonium chloride.

Chlorhexidine [CHX] is a widely used broad-spectrum disinfectant for prepared tooth structure. Chlorhexidine digluconate is effective against both Gram-positive and Gram-negative bacteria [4]. Its cationic composition binds to negatively charged carboxyl and hydroxyl groups of collagen and non-collagenous phosphoproteins in demineralized dentin. It also electrostatically interacts with phosphate groups of hydroxyapatite crystallites in mineralized dentin. At higher concentrations, it leads to cytoplasmic congealing by coagulation of intracellular components. Although it removes the loose smear debris, it preserves the hybrid layer [5]. Researchers recommend CHX application on etched dentin before the bonding procedure [6]. Researchers reported its bonding to the etched dentin to be higher than the mineralized dentin [7]. However, the effects of application before the bonding procedure and its ramifications in the self-etching bonding protocol need further evaluation.

Sodium hypochlorite (NaOCl) is commonly used as an endodontic irrigant for chemical debridement of root canal space. It is also utilized as a cavity disinfectant because of its antibacterial effect and enhanced adhesive wettability. Furthermore, it deproteinizes both demineralized and mineralized dentin. However, its dentin deproteinization effect on bond strength is contentious. Laie et al. [8] reported the unfavorable consequence of deproteinization and strong oxidizing with reduced bond strength. Meanwhile, Hayashi et al. [9] found enhanced bond strength due to improved resin infiltration. NaOCl alters the organic–inorganic ratio by removing the organic constituents mainly from the collagen matrix [10]. Ethylenediaminetetraacetic acid (EDTA) mild chelator achieves moderate dentine demineralization by removing the smear layer [11]. It avoids the denaturation of collagen and maintains the hybrid layer quality due to the existence of residual hydroxyapatite crystals within the collagen matrix [12]. Further, 2% iodine is typically used as a plaque disclosing and disinfection solution. Iodine, being a small molecule, rapidly penetrates microorganisms and oxidizes critical proteins, nucleotides, and fatty acids, leading to cell death [13]. Hence, the iodine solution could be a suitable dentin-enamel disinfectant before restorative procedures. However, its interference in adhesive monomer wetting and polymerization could affect the bonding strength of dental adhesives.

Various chemical disinfectant agents can alter the organic and inorganic component’s proportion of dentin [14], thereby compromising the structural properties such as microhardness, solubility, and permeability of dentin substrate. Microhardness tests provide evidence of changes in mineral content, which could influence the bonding potential of the dentin surface [15]. Previous studies showed the varying effect of chemical irrigants on the microhardness of radicular dentin [16,17]. Previous researchers reported the alteration in dentin microhardness resulting in the corresponding variations in micro-shear bond strength potential [18,19]. The total-etch approach with the application of 37 wt.% phosphoric acid yields the different structural properties of dentin compared to the self-etch adhesive protocol. Hence, the effect of these chemical disinfectants on bonding strength and microhardness is expected to be distinct. Additionally, concurrent evaluation of microhardness and shear bond strength in different bonding techniques will enhance knowledge of the correlation between the chemical disinfectant and the bonding performance. Thus, this study aimed to assess the effect of chlorhexidine digluconate, sodium hypochlorite, ethylenediaminetetraacetic acid and povidone iodine disinfection on the micro-shear bond strength and microhardness of adhesive resin-infiltrated dentin. The null hypothesis was that different antibacterial agents do not affect the shear bond strength and microhardness of resin-infiltrated dentin substrate.

## 2. Materials and Methods

### 2.1. Teeth Sample Preparation

**Figure 1:**
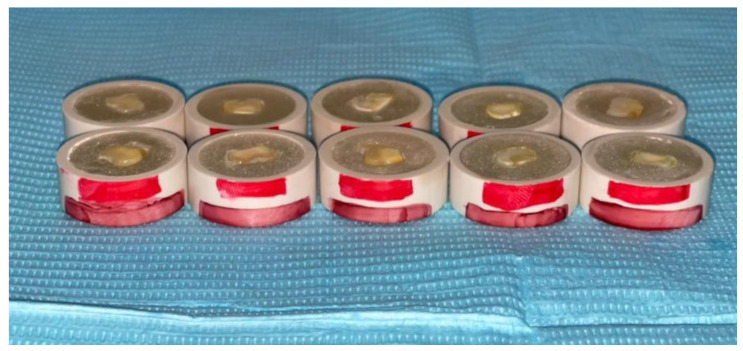
Teeth samples prepared for surface disinfection and bonding procedure.

A total of 100 intact third molar teeth were collected from the oral surgery department. Sample teeth were extracted for therapeutic reasons and due consent from the patients was obtained for utilizing their teeth for research purposes. The institutional review board, (College of Dentistry, King Khalid University, KSA (IRB/KKUCOD/ETH/2021-22/017) approved the research protocol. Teeth samples were cleaned of calculus and soft tissues with hand scaling, disinfected with 7-day immersion in 10% formalin solution and stored in distilled water at room temperature until the preparation for the study. The root and cuspal portions of teeth were sectioned at a cementum–enamel junction and central fossae depth using a low-speed diamond disk saw (Isomet, Buehler Ltd., Lake Bluff, IL, USA) under water coolant. Teeth samples were further sectioned vertically into buccal and lingual halves under water coolant. Resultant 200 sectioned halves were embedded into autopolymerizing clear polymethyl methacrylate acrylic resin (Major.Base.20, Major Prodotti Dentari S.p.A., Moncalieri, Italy). The samples were implanted horizontally with their axial surfaces parallel to an outer surface of resin with the help of a vertical holding machine. The underlying dentin surface was exposed and flattened with sequential grinding with 400-, 600-, and 800-grit waterproof SiC paper discs (Figure 1). Between each grinding stage, the samples were cleaned with the normal saline solution under ultrasonic cleaner. The dentin surface flattening and smear layer was standardized by using each disc for 1 min by a single operator. Teeth samples were randomly divided into five groups (*n* = 40) according to a disinfectant protocol used for surface treatment. Each main group was subdivided into two subgroups (*n* = 20) according to the adhesive protocols of total-etch and self-etch. Out of these 20 samples, 10 samples were utilized for micro-shear bond strength, and the remaining 10 samples were used for the microhardness test. A sample size of 10 for each subgroup was estimated according to the previously published studies [20,21]. The sample size was calculated using G* Power software (version 3.1; University of Dusseldorf), with an effect size (d) of 1.4, α of 0.05, and 1-β (power) of 0.85 [22].

### 2.2. Surface Disinfectant Groups

Group I—Control: teeth samples were devoid of any chemical surface disinfection, and adhesive protocol of total-etch and self-etch was followed as per the manufacturer’s instruction.

The experimental groups were disinfected with the following active chemical ingredient.

Group II—Chlorhexidine Digluconate (CHX): 2% CHX (Consepsis, Ultra dent, South Jordan, UT, USA) was applied to the dentin surface with a micro brush and left in contact with the dentin surface for 30 s. Excess CHX was blot-dried followed by light air-drying for 15 s.

Group III—Sodium Hypochlorite (NaOCl) (Sultan Health care, York, PA, USA): Dentin surface was treated with 5.25% NaOCl for 60 s, subsequently water was rinsed for 60 s and gently air-dried.

Group IV—Ethylenediaminetetraacetic acid (EDTA): 17% EDTA (m.me, PO box 251395, Dubai, United Arab Emirates), the gel was applied onto the dentin surface for 60 s, followed by water rinsing for 15 s and blot-dried.

Group V—Povidone Iodine: Povidone iodine (10%) was applied using applicator tips on an exposed dentin surface for 60 s and subsequently lightly air-dried.

### 2.3. Adhesive Procedures

Each surface disinfection group was divided into two groups (*n* = 20) to be bonded with total-etch and self-etch bonding protocols. The teeth samples that followed the total-etch bond were initially etched with 37% phosphoric acid and subsequently disinfected with chemical agents according to the groups. The teeth samples bonded with the self-etch protocol were disinfected with a chemical disinfectant at the beginning and later self-etch adhesives were applied to the dentin substrate. The teeth samples for total-etch groups (STAE, SDI, Bayswater, Victoria, Australia) were initially etched with 37% phosphoric acid (Total Etch, Ivoclar Vivadent Inc. Amherst, NY, USA) for 15 s, afterward gently water-rinsed for 20 s and blot-dried with cotton pellet. The adhesive was applied to saturate the dentin surface, gently air-dried from oil-free air for 2 s, and light-cured for 10 s at 800 mW/cm^2^ light intensity. The teeth sample bonded with the self-etch protocol, universal bonding agent (Prime & Bond Universal, Dentsply DeTrey GmbH, Konstanz, Germany) was applied onto the dentin, the bonding agent was agitated with a micro brush for 20 s, gently air-dried for 5 s and light-cured for 20 s.

The adhesive impregnated dentin surface of 10 samples from each subgroup was restored with a nano-hybrid composite (Tetric N-Ceram, Ivoclar Vivodent AG, Schaan, Liechtenstein) cylinder measuring 3 mm diameter × 2 mm height. The composite resin dimension was standardized with silicone putty with cylindrical space corresponding to the dentin surface. Packed composite resin was covered with a flat glass slab and light-cured with 700 mW/cm^2^ light-emitting diode light-curing unit (Bluephase, Ivoclar Vivadent, Amherst, NY, USA) for 20 s.

### 2.4. Micro-Shear Bond Strength and Microhardness Testing

**Figure 2:**
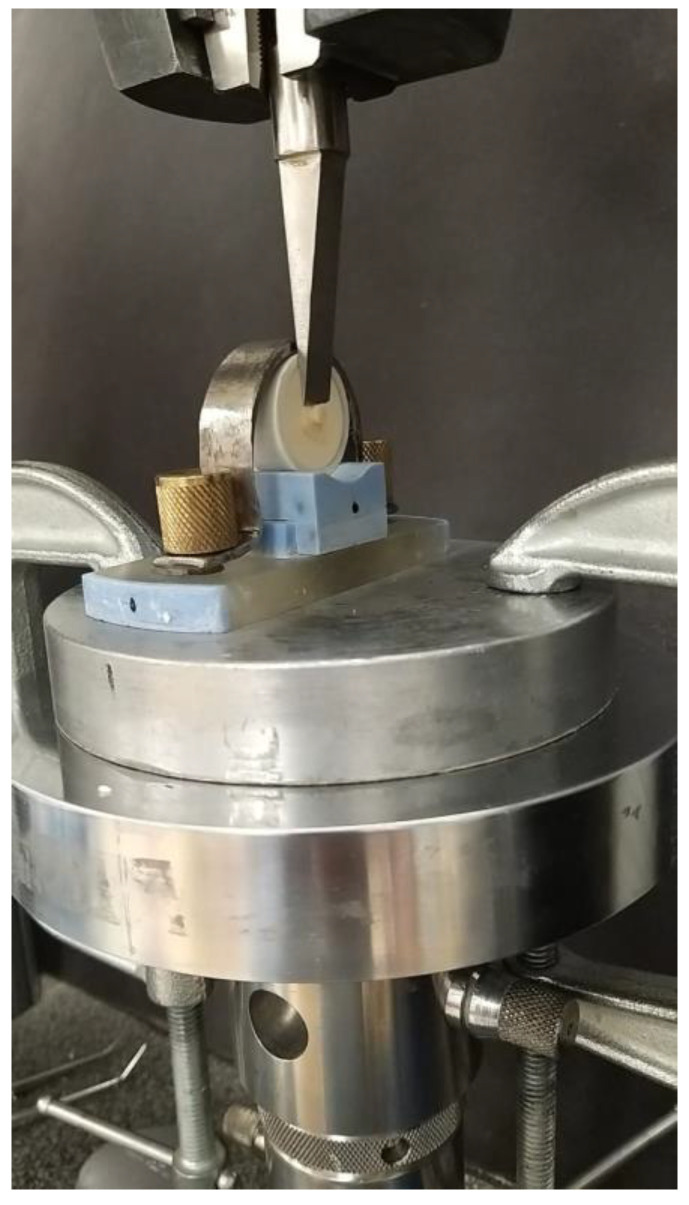
Micro-shear bond strength evaluation with INSTRON.

The micro-shear bond strength (µSBS) of samples was tested according to the ISO/TS 11405:2015 specification. Specimens with bonded composite restoration were stored in 37 °C distilled water for 24 h, followed by 12,000 thermal cycles (Thermocycler, SD Mechatronik, Feldkirchen-Westerham Germany) between 5–55 °C with a 30 s dwelling time. Subsequently, the bonded composite–dentin interface was subjected to shear stress with a 200-µM chisel-shaped head with a ramp rate of 1 mm/min (Figure 2). The maximum load at fracture was recorded in Newton (N). The debonded interface was assessed with a digital microscope (Hirox, Hackensack, NJ, USA) at ×25 magnification to categorize the mode of failure as adhesive, cohesive, or mixed. The failures recorded at the composite–dentin interface were categorized as an adhesive failures, while the failures within composite resin were recorded as cohesive failures. Failures at combined locations of the composite–dentin interface and within the composite resin were considered mixed failures.

**Figure 3:**
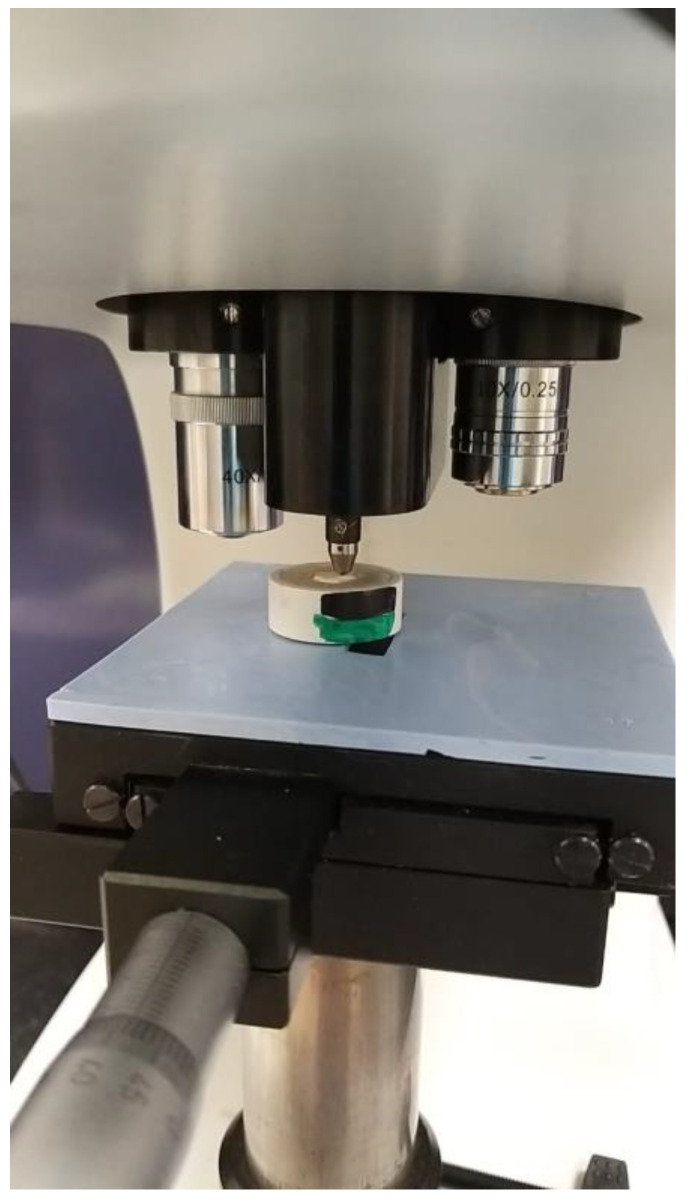
Microhardness testing on the teeth sample.

The samples intended for microhardness assessment were tested using a micro-indenter instrument (FALCON-500, INNOVATEST Europe BV, Borgharenweg, Maastricht, NL, Canada). Adhesive resin-infiltrated dentin surface was evaluated at three different locations by Vickers digital micro-indenter with 200 gf (gram force) and a retention time of 15 s (Figure 3).

### 2.5. Statistical Analysis

Statistical analysis was performed using SPSS 19 software (IBM Corporation, Armonk, NY, USA). The data were evaluated by one-way ANOVA and Tukey HSD post hoc tests. The level of statistical significance was considered to be 0.053.

## 3. Results

The micro-shear bond strength of various disinfectants in the total-etch and self-adhesive systems over 24 h and thermocycling are summarized in Table 1. Both chemical disinfectant type and adhesive system significantly affected the bond strength. Total-etch adhesive groups displayed higher shear bond strength compared to self-etch bond counterpart groups. Control groups in total-etch and self-etch adhesive protocols showed the higher µSBS in their respective groups with 177.55 (6.27) N and 144.08 (3.68) N, respectively. Amongst the chemical disinfectants evaluated in the study, 2% CHX showed the highest µSBS in both total-etch and self-etch groups with 174.28 (7.28) N and 137.73 (7.51) N. NaOCl followed it with corresponding values at 155.06 (9.95) N and 130.10 (7.12) N. The groups treated with EDTA showed moderate µSBS in both total-etch and self-etch groups with 146.28 (6.05) N and 114.91 (7.39) N, respectively. Povidone iodine disinfection displayed the least µSBS for both total-etch (124.05 N) and self-etch (96.80) adhesive protocols. 

All disinfection regimens evaluated in the study in both bonding protocols decreased the mean hardness of dentin compared to the control group. Contradictory to µSBS values, self-etch groups showed higher dentin microhardness compared to total-etch counterparts (Table 2). During dentin microhardness testing, 2% CHX disinfection groups displayed the highest values for both total-etch and self-etch adhesive groups with corresponding values of 59.18 (1.00) MV and 63.31 (1.94) HV compared to corresponding values of 69.24 (3.10) HV and 71.47 (3.43) HV from the control group. Dentin substrate treated with povidone iodine also had moderately higher microhardness in total-etch adhesives (54.02 HV) and self-etch groups (60.24 HV). The EDTA group recorded the lowest dentin microhardness in total-etch adhesives with 31.11 (1.04) HV; NaOCl disinfectant amongst self-etch adhesive was at 39.18 (1.23) HV.

A one-way ANOVA (Table 3) was conducted to compare the effect of different dentin disinfection on the micro-shear bond strength. One-way ANOVA analysis exhibited the effect of various disinfectants on the micro-shear bond strength was significant in the total-etch adhesive protocol, F (68.918) = 43420581, *p* = 0.000. A comparable significant difference in micro-shear bond strength was observed in the self-etch adhesive technique, F (56.370) = 3268.01, *p* = 0.000. The effect of different surface disinfection on dentin microhardness was also statistically significant in total-etch adhesives F (2202.981) = 1499.107, *p* = 0.000, and self-etch group with F (1103.945) = 2226.228, *p* = 0.000.

The Shapiro–Wilk test for normality confirmed the normal data distribution with *p* > 0.05 in different disinfection groups for both the total-etch and self-etch groups. Tukey HSD post hoc multiple comparison tests (Table 4) showed a significant difference between micro-shear bond strength of all disinfectant groups except between control and CHX (*p* = 0.873) in total-etch, control and CHX in self-etch groups (*p* = 0.270).

The CHX in the total-etch group showed a similar failure mode to the control group with six cohesive and four mixed failures (Table 5). A higher number of adhesive failures was observed in EDTA and povidone iodine groups in both total-etch and self-etch bonding protocols.

## 4. Discussion

After failed attempts for remineralization, the definitive treatment for dental caries is to remove the infected tooth substrate and rehabilitate it by either direct or indirect restorations. Regardless of meticulous efforts to eliminate the infected tissues and bacterial contents, multiple studies [22,23] found that 20–25% of teeth still contain viable bacteria in the deepest portion of the carious lesion. This leads to rehabilitation failures such as a fractured tooth and secondary caries, besides bonding degradation [24,25]. Consequently, the disinfection of the prepared tooth is an essential step before restorative procedures. However, despite its advantages, the impact of various disinfectant compounds on the adhesion and microhardness, especially in different bonding protocols, is not clear. This in vitro study explored the effect of various disinfection compounds on the bonding strength in total-etch and self-etch bonding protocols. The results showed the disinfection chemical compound impacted the shear bond strength and dentin microhardness. Hence, the null hypothesis that chemical disinfectant would not affect adhesion and dentin microhardness was rejected.

Study results showed that the total-etch bonding technique had better shear bond strength across all the disinfection protocols compared to self-etch groups. The results were consistent with the findings of earlier studies [26,27,28,29]; they reported the higher shear bond strength of total-etch adhesive than self-etching adhesives. Total-etch adhesives remove the smear layer and dissolved minerals during water rinsing. Meanwhile, self-etch adhesives depend on the simultaneous demineralization and infiltration of acidic monomer within the tooth substrate. Kerby, R.E. [30] found shorter resin tags in self-etch adhesive compared to phosphoric acid conditioned dentin structure from total-etch adhesives. Villela-Rosa et al. [31] concluded the shear bond strength of dentin largely depends on substrate depth and adhesive/depth interaction. Water blisters within polymerized hydrophilic resin monomers such as hydroxyethyl methacrylate could contribute to a weak adhesive interface in self-etch adhesive [32].

Study results reconfirm the reports from earlier reports of a negative, deleterious effect of chemical disinfection agents on the bonding of dentin substrate to various extents [33,34]. Among the chemical disinfectants assessed in the study, CHX demonstrated the highest micro-shear bond strength in both TE and SE Adhesive techniques at 174.28 N and 137.73 N, respectively, with no significant reduction in μSBS. The results were in agreement with the observations of Say et al. [35] and Campos et al. [36]. They reported no significant reduction of μSBS in both etch-and-rinse and a universal system. Lenzi et al. [37] also concluded that CHX did not influence the immediate bond strength to sound or caries-affected dentin of primary and permanent teeth. Bravo et al. [38] also compared the three different adhesive systems—etch-and-rinse, self-etch and universal, and reported no differences between them. Many researchers hypothesized the influence of CHX disinfection on bonding strength. Meiers et al. [39] showed the removal of loose smear debris and facilitating penetration of acidic monomer in their scanning electron microscopic study. Meanwhile, Perdigao et al. [40] postulated strong positive ionic-charge binding to phosphate groups on the dentin surface, leading to increased dentin surface energy and enhanced primer wettability.

NaOCl is the most frequently used cavity disinfectant due to its well-recognized antibacterial action and aiding wettability. The results show the negative influence on the bond strength in both total and universal bonding protocols. Mohammad et al. [41] showed that the use of NaOCl decreased the shear bond strength of both fifth- and seventh-generation adhesive resins. Ercan et al. [20] reported that the NaOCl disinfection decreased the bond strength in the self-etching bonding system, whereas no adverse effect was observed with the etch-and-rinse adhesive. Santos et al. [42] reported a significant reduction in bond strength from NaOCl solution. However, the result contradicted Elkassas et al. [43] and Fawzy [44], who reported the NaOCl pretreatment increased the bond strength of the self-etch adhesive. The contradictory results could be because of differences in experimental protocols, adhesive agents, and dentin substrate. A decrease in bond strength might be attributed to the oxygen release after the disintegration of NaOCl into NaCl and O2 [45]. The free-residual free radicals might hinder the vinyl-free radical proliferation during light activation of the adhesive system, ensuing premature chain termination and incomplete polymerization [8].

Ethylenediamine tetraacetic acid (EDTA) is carboxylic acid containing organic compounds. It can chelate the calcium ions and selectively remove the hydroxyapatite without altering the fibrillar structure of the collagen network and collagen denaturation [46]. The EDTA surface treatment negatively affected the bond strength in both total-etch and universal adhesives. Findings of the earlier research are contradictory: Wang et al. [47] reported a decrease in bond strength with the use of 15% EDTA, while Osorio et al. [12] and Kasraei et al. [21] reported an increase in mean bond strength in different adhesive systems. The reason for variations in the results could be due to EDTA pH, application time, and concentration. Moreover, 1.5–5% concentration is ineffective in removing smear layer, while 15% concentration with prolonged condition time leads to complete dissolution of smear layer, substantial widening of tubule orifice, and depth of demineralization [48]. The teeth sample treated with povidone iodine recorded significantly lower µSBS compared with the control group. The results concur with the findings of Silva et al. [49], who reported a considerable reduction in bond strength for ethanol and/or water-based adhesives. Suma et al. [50] recorded that the use of 0.3% iodine with self-etch adhesive led to significantly lower SBS of composite to resin. The residual chemical molecules could inhibit the wettability of adhesive resins and consequently compromise the ability to impregnate the dentin substrate [51].

Structural properties, including the microhardness of dentin, may alter after a chemical disinfection procedure. This could be due to a change in the proportion of organic and inorganic components [14]. Microhardness is regarded as evidence of mineral alterations; it could influence the bonding potential of the dentin surface. Amongst the disinfectant agents evaluated, NaOCl and EDTA experimental groups showed a substantial reduction in microhardness in both total-etch and universal adhesive protocols. Earlier studies [16,52] reported the NaOCl and EDTA solution negatively affected the microhardness of radicular dentin. Earlier studies showed dissolution of the main organic component of dentin Type I collagen components by NaOCl, thereby reducing the modulus of elasticity and flexural strength of dentin. It is also known to dissolve inorganic components such as magnesium and phosphate ions, while increasing dentinal carbonate content [53]. Doğan et al. [54] recorded a considerable variation in Ca/P ratio after NaOCl treatment. Hence, the alteration of mineral and organic contents is attributed to a reduction of dentin microhardness. The strong chelating effect of EDTA by the dissolution of calcified components would lead to softening and a reduction in microhardness of dentin [15]. Study results showed the marginal reduction of microhardness in CHX experimental groups. Previous studies showed the effect on structural properties depends on the contact time and concentration of CHX [17]. Naenni et al. [55] observed a lack of tissue dissolution capacity and removing smear layer by CHX. Hence, the remaining smear layer acts as a potential barrier to minimizing the irrigant’s contact time with dentin. However, at a higher concentration of 2% and longer contact time, CHX has reported a decrease in Ca/P proportion and microhardness [56]. The lack of ability to dissolve necrotic tissues and chemical remnants post-surface treatment could result from a marginal reduction in the dentin microhardness in the povidone-iodine experimental group.

Clinical implications of the study results include that clinicians should thoroughly consider the effect of various disinfectant agents on the bonding potential and structural integrity of dentin substrate. Considering the study results, CHX could be considered a safe choice as a disinfectant for a cavity or prepared tooth structure.

Limitations of the study included that the study considered only the immediate µSBS between composite and tooth structure. Additionally, the study evaluated the chemical disinfectants in limited concentration. The study protocol is in vitro. Hence, due diligence is required to extrapolate the results to a clinical situation. Further studies are suggested to evaluate the long-term bond-strength sustainability. We also recommend future studies to assess the effect of these chemical disinfectants on bond strength and microhardness in infected and sclerotic dentin.

## 5. Conclusions

Within the limitations of the study, the following conclusions were drawn:

1 All the chemical disinfectants assessed in the study showed a varying amount of negative effects on both bond strength and microhardness of dentin.
2 Total-etch adhesive protocol showed higher µSBS values in most of the groups compared to self-etch adhesives.
3 The application of CHX resulted in an insignificant effect on the µSBS values and a marginal reduction in the microhardness of dentin.
4 Although NaOCl surface treatment had a lesser impact on µSBS compared to EDTA and povidone iodine, it caused a substantial reduction in microhardness in both total-etch and self-etch adhesives.

## Figures and Tables

**Table 1:** Mean micro-shear bond strength (N) of different surface disinfectants in total-etch and self-etch bonding groups.

Disinfection Agent	Adhesive Protocol	
	Total-Etch	Self-Etch
Control	177.55 (6.27)	144.08 (3.68)
Chlorhexidine	174.28 (7.28)	137.73 (7.51)
Sodium hypochlorite	155.06 (9.95)	130.10 (7.12)
EDTA	146.28 (6.05)	114.91 (7.39)
Povidone iodine	124.05 (7.95)	96.80 (8.36)

**Table 2:** Mean microhardness (MV) of dentin surface amongst different surface disinfectants in total-etch and self-etch bonding groups.

Disinfection Agent	Adhesive Protocol	
	Total-Etch	Self-Etch
Control	69.24(3.10)	71.47(3.43)
Chlorhexidine	59.18(1.00)	63.31(1.94)
Sodium hypochlorite	50.14(0.58)	39.18(1.23)
EDTA	31.11(1.04)	47.59(1.15)
Povidone iodine	54.02(0.52)	60.24(1.20)

**Table 3:** One-way ANOVA analysis of different surface disinfection group for mean Micro-Shear bond strength and microhardness.

GROUP	Testing Parameter	SOURCE	df	SS	MS	F	*p*
TOTAL ETCH	Micro-Shear bond strength	Between the Groups	4	19,133.64	4783.41	82.070	0.000 *
		Within Groups	45	2622.802	58.284		
		Total	49	21,756.445			
	Microhardness	Between the Groups	4	7901.849	1975.462	799.800	0.000 *
		Within Groups	45	111.148	2.470		
		Total	49	8012.996			
SELF ETCH	Micro-Shear bond strength	Between the Groups	4	14,486.829	3621.707	73.77	0.000 *
		Within Groups	45	2209.256	49.06		
		Total	49	16,696.085			
	Microhardness	Between the Groups	4	6638.552	1659.638	418.373	0.000 *
		Within Groups	45	178.510	3.967		
		Total	49	6817.062			

* The mean difference is significant at the 0.05 level.

**Table 4:** Tukey HSD post hoc multiple comparison tests of different surface disinfection group for mean micro-shear bond strength and microhardness.

GROUP	Testing Parameter	(I) Group	(J) Group				
			Control	CHX	NaOCl	EDTA	IOD
TOTAL ETCH	Micro-Shear bond strength	Control		0.873	0.000 *	0.000 *	0.000 *
		CHX	0.873		0.000 *	0.000 *	0.000 *
		NaOCl	0.000 *	0.000 *		0.093	0.000 *
		EDTA	0.000 *	0.000 *	0.093	0.000 *	0.000 *
		IOD	0.000 *	0.000 *	0.000 *	0.000 *	
	Microhardness	Control		0.000 *	0.000 *	0.000 *	0.000 *
		CHX	0.000 *		0.000 *	0.000 *	0.000 *
		NaOCl	0.000 *	0.000 *		0.000 *	0.000 *
		EDTA	0.000 *	0.000 *	0.000 *		0.000 *
		IOD	0.000 *	0.000 *	0.000 *	0.000 *	
SELF ETCH	Micro-Shear bond strength	Control		0.270	0.000 *	0.000 *	0.000 *
		CHX	0.270		0.124	0.000 *	0.000 *
		NaOCl	0.000 *	0.124		0.000 *	0.000 *
		EDTA	0.000 *	0.000 *	0.000 *		0.000 *
		IOD	0.000 *	0.000 *	0.000 *	0.000 *	
	Microhardness	Control		0.000 *	0.000 *	0.000 *	0.000 *
		CHX	0.000 *		0.000 *	0.000 *	0.000 *
		NaOCl	0.000 *	0.000 *		0.000 *	0.010 *
		EDTA	0.000 *	0.000 *	0.000 *		0.000 *
		IOD	0.000 *	0.000 *	0.010 *	0.000 *	

* The mean difference is significant at the 0.05 level.

**Table 5:** Fracture mode recorded for each group (in number).

Group	Total-Etch			One-Step Self-Etch		
	**Adhesive**	**Cohesive**	**Mixed**	**Adhesive**	**Cohesive**	**Mixed**
Control	0	6	4	0	5	5
Chlorhexidine	0	6	4	1	4	5
Sod hypochlorite	1	3	6	1	4	5
EDTA	2	2	6	3	1	6
Povidone iodine	4	1	5	5	1	4

## Data Availability

Data sharing not applicable.
